# MiR-126-3p inhibits apoptosis and promotes proliferation by targeting phosphatidylinositol 3-kinase regulatory subunit 2 in porcine ovarian granulosa cells

**DOI:** 10.5713/ajas.19.0290

**Published:** 2019-08-26

**Authors:** Xiaofeng Zhou, Yingting He, Yao Jiang, Bo He, Xi Deng, Zhe Zhang, Xiaolong Yuan, Jiaqi Li

**Affiliations:** 1 Guangdong Provincial Key Lab of Agro-Animal Genomics and Molecular Breeding, National Engineering Research Centre for Breeding Swine Industry, College of Animal Science, South China Agricultural University, Guangzhou, Guangdong 510642, China

**Keywords:** Ovarian Granulosa Cells, miR-126-3p, Phosphatidylinositol 3-Kinase Regulatory Subunit 2 (*PIK3R2*), Cell Apoptosis, Cell Proliferation

## Abstract

**Objective:**

Numerous studies have indicated that the apoptosis and proliferation of granulosa cells (GCs) are closely related to the normal growth and development of follicles and ovaries. Previous evidence has suggested that miR-126-3p might get involved in the apoptosis and proliferation of GCs, and phosphatidylinositol 3-kinase regulatory subunit 2 (*PIK3R2*) gene has been predicted as one target of miR-126-3p. However, the molecular regulation of miR-126-3p on *PIK3R2* and the effects of *PIK3R2* on porcine GCs apoptosis and proliferation remain virtually unexplored.

**Methods:**

In this study, using porcine GCs as a cellular model, luciferase report assay, mutation and deletion were applied to verify the targeting relationship between miR-126-3p and *PIK3R2*. Annexin-V/PI staining and 5-ethynyl-2′-deoxyuridine assay were applied to explore the effect of *PIK3R2* on GCs apoptosis and proliferation, respectively. Real-time quantitative polymerase chain reaction and Western Blot were applied to explore the regulation of miR-126-3p on *PIK3R2* expression.

**Results:**

We found that miR-126-3p targeted at *PIK3R2* and inhibited its mRNA and protein expression. Knockdown of *PIK3R2* significantly inhibited the apoptosis and promoted the proliferation of porcine GCs, and significantly down-regulated the mRNA expression of several key genes of PI3K pathway such as insulin-like growth factor 1 receptor (*IGF1R*), insulin receptor (*INSR*), pyruvate dehydrogenase kinase 1 (*PDK1*), and serine/threonine kinase 1 (*AKT1*).

**Conclusion:**

MiR-126-3p might target and inhibit the mRNA and protein expressions of *PIK3R2*, thereby inhibiting GC apoptosis and promoting GC proliferation by down-regulating several key genes of the PI3K pathway, *IGF1R*, *INSR*, *PDK1*, and *AKT1*. These findings would provide great insight into further exploring the molecular regulation of miR-126-3p and *PIK3R2* on the functions of GCs during the folliculogenesis in female mammals.

## INTRODUCTION

Granulosa cells (GCs) have been shown to play an important role in the dynamic process of folliculogenesis, including follicular initiation [1], dominance [2], ovulation [3], luteinization [4] and atresia [5,6] by regulating proliferation, cell cycle, apoptosis, and synthesis of steroid hormones [7] in female mammals. These processes are important to the maturation of oocyte and propagation of species. Previous studies have reported that the estrogen secreted by GCs is reduced in estrogen receptor 2 mutant mice, resulting in impaired follicle maturation and failure of ovulation [8–10], and moreover, the high apoptosis ratio of GCs could impair folliculogenesis and give rise to the increase follicular atresia [11,12]. These observations show that GCs are closely related to the development of follicles and ovaries.

The microRNA is a class of single-stranded non-coding RNA consisting of about 22 nucleotides in length that regulates the expression of the target gene at the post-transcriptional level [13]. Studies have shown that a variety of miRNAs are involved in the development of ovarian follicles, corpus luteum formation, degeneration as well as follicular atresia [14,15]. For example, in mouse GCs, miR-224 targets SMAD family member 4 (*Smad4*) to promote the proliferation of GCs and the expression of aromatase cytochrome P450, family 19, subfamily a, polypetide1 (*Cyp19a1*) through the transforming growth factor, beta 1 (*TGFβ1*)/Smads pathway, thereby modulating the secretion of estradiol [16]. In pigs, miR-26b was differentially expressed between normal and atresia follicles, and has been proved to promote GCs apoptosis by targeting DNA damage related genes ATM serine/threonine kinase (*ATM*) [17,18]. In bovine, miR-378 may suppress the apoptosis of luteal cells by targeting interferon gamma receptor 1 gene [19]. However, the molecular mechanisms of how miRNAs regulate GCs proliferation and apoptosis remain unclear in mammals.

A recent study has reported that miR-126-5p is significantly decreased in GCs of polycystic ovarian syndrome (PCOS) patients, compared to healthy women, and may be involved in GC apoptosis [20]. In our previous study, we have proved that miR-126-3p inhibits apoptosis and promotes proliferation of porcine ovarian GCs [21]. These results suggested that miR-126-3p might exhibit an essential role in apoptosis and proliferation of porcine GCs, and consequently give rise to dynamic impact on the development of follicles. In this study, we found that phosphatidylinositol 3-kinase regulatory subunit 2 (*PIK3R2*) was a potential target of miR-126-3p by using bioinformatic algorithms. *PIK3R2* is a member of phosphatidylinositol kinase (PI3K) 3-family gene [22]. It is reported that *PIK3R2* can encode p85β, an enzyme that generates 3-polyphosphoinositides at the plasma membrane, to inhibit phosphorylation of Akt and resulting in suppressing PI3K/AKT proliferation-survival signaling pathway [23,24]. In rheumatoid arthritis synovial fibro-blasts cell, *PIK3R2* inhibited the proliferation and promoted the apoptosis by regulating PI3K/AKT pathway [25]. However, the function of *PIK3R2* in porcine GCs has not been reported. Therefore, we hypothesized that miR-126-3p might target *PIK3R2* to regulate proliferation and apoptosis of porcine ovarian GCs. In this study, we aimed to investigate whether miR-126-3p targeted and repressed the expression of *PIK3R2*, and consequently regulated the proliferation and apoptosis of porcine ovarian GCs.

## MATERIALS AND METHODS

### Ethics approval

All experiments conducted in this study strictly followed the guidelines of the Animal Care and Use Committee of South China Agricultural University Guangzhou, China (approval number: SCAU#2013-10).

### Ovarian granulosa cell culture and transient transfection

**Figure 1:**
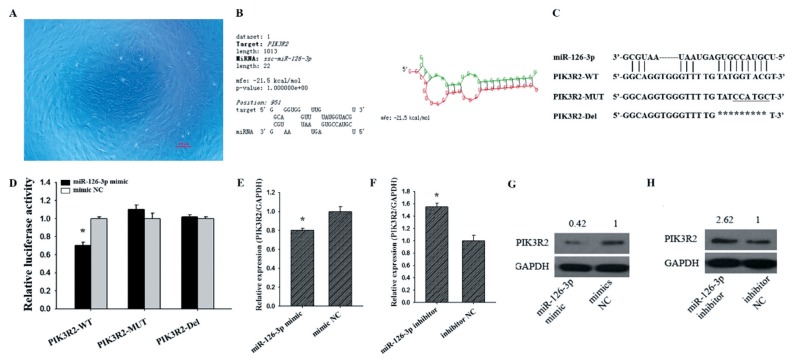
*PIK3R2* is a target gene of miR-126-3p. The cultured porcine GCs for 24 h (A). A miR-126-3p binding site within the *PIK3R2* 3′-UTR is predicted by RNAhybrid (B). Wild-type sequence (*PIK3R2*-WT), mutated sequence (*PIK3R2*-MUT), and deleted sequence (*PIK3R2*-Del) of the miR-126-3p binding site (C). Relative luciferase activities of *PIK3R2*-WT, *PIK3R2*-MUT, and *PIK3R2*-Del after transfection with the miR-126-3p mimics or mimic NC in GCs (D). Relative mRNA expression of *PIK3R2* after transfection with the miR-126-3p mimics (E) and inhibitors (F) in GCs. Relative protein expression of *PIK3R2* after transfection with the miR-126-3p mimics (G) and inhibitors (H) in GCs. The scale bar of the micrograph is 100 *μ*m. *PIK3R2*, phosphatidylinositol 3-kinase regulatory subunit 2; GCs, granulosa cells; GAPDH, glyceraldehyde 3-phosphate dehydrogenase. * indicates p<0.05. Mimic NC is the negative control of miR-126-3p mimic and inhibitor NC is the negative control of miR-126-3p inhibitor. The relative protein expression level of *PIK3R2* was normalized by GAPDH values.

The ovaries of pre-puberty sows were collected from a local slaughterhouse and transported to the laboratory using phosphate buffered saline (PBS) containing penicillin (100 IU/mL) and streptomycin (100 μg/mL) (Invitrogen, Shanghai, China). Subsequently, the GC was aspirated by inserting a syringe into a 3 to 5 mm follicle, and the separated GCs were washed twice with PBS. The cells were then seeded into culture flasks containing 10% fetal bovine serum (Hyclone, Logan, UT, USA) in Dulbecco’s modified eagle medium (DMEM) (Hyclone, USA) and 100 IU/mL penicillin, 100 μg/mL streptomycin, and finally incubated at 37°C under 5% CO_2_. After 24 h of culturing GCs, the cell confluence reached 70% to 90% for transfection (Figure 1A). The GCs were transfected with miR-126-3p inhibitor, inhibitor negative control (NC), siRNA-*PIK3R2*, or siRNA-NC for 48 h using Lipofectamine 3000 Reagent (Invitrogen, China).

### Real-time quantitative polymerase chain reaction analysis

TRIzol reagent (TaKaRa, Tokyo, Japan) was used to extract total RNA from the sample, and then the RevertAid First Strand cDNA Synthesis Kit (Thermo Scientific, Waltham, MA, USA) was used to reverse-transcribe the mRNAs. Maxima SYBR Green qRT-PCR Master Mix (2x) (Thermo Scientific, USA) was used to quantify the relative expression levels of mRNAs in the LightCycler real-time polymerase chain reaction (PCR) system. Using the expression level of glyceraldehyde 3-phosphate dehydrogenase (GAPDH) as endogenous control, the relative expression level of *PIK3R2* was calculated with the 2−ΔΔct method. The Primer sequences are listed in Table 1.

### Cell proliferation and apoptosis assay

The Cell-LightTM 5-ethynyl-2′-deoxyuridine (EdU) Apollo 567 In Vitro Kit (RiboBio Co., Ltd., Guangzhou, China) was used to analyze cell proliferation. Briefly, GCs were cultured in 48-well plates and transfected with plasmid for 36 h. The GCs were incubated at room temperature with 50 μM EdU for 2 h, washed twice with PBS, and then incubated with 80% acetone for 30 min. After GCs were washed twice with PBS, 0.5% Triton X-100 was added for 10 min, 1×Apollo was incubated in darkness for 30 min, and Hoechst was incubated for 30 min. Finally, three fields were randomly selected from each well and GCs were counted under an inverted fluorescence microscope.

The Annexin V-FITC Apoptosis Detection Kit (BioVision, Milpitas, CA, USA) was used to analyze cell apoptosis. Briefly, GCs were cultured in 6-well and transfected with plasmids for 48 h. The collected cells were centrifuged at 1,000 rpm for 5 min, supernatant discarded, and washed twice with PBS. Then, 500 μL of 1X Annexin V buffer was added to gently resuspend the cells, and 5 μL of Annexin V-FITC and 5 μL of propidium iodide staining solution were added and mixed. Finally, flow cytometry was performed after incubation for 15 min at room temperature in darkness. For results, the figure has four quadrants, the lower right quadrant is annexin-positive/PI-negative early apoptotic cells, the upper right quadrant is annexin-positive/PI-positive late apoptotic cells, the lower left quadrant is living cell, and the upper left quadrant is mechanical injury cells. In this study, the apoptotic ratio of GCs is the sum of early and late apoptosis.

### Vector construction and dual-luciferase reporter assay

The region of *PIK3R2* gene 3′-UTR that contains a potential binding site of miR-126-3p was cloned and ligated it to the pmirGLO dual luciferase miRNA target expression vector (Promega, Madison, WI, USA). We constructed three recombinant vectors, the recombinant vector containing the wild-type miR-126-3p binding site sequence was named as *PIK3R2*-wild-type (WT), the mutant vector containing part of the miR-126-3p binding site sequence was named as *PIK3R2*-mutant, and the recombinant vector deleting part of the miR-126-3p binding site sequence was named as *PIK3R2*-deleted (Del). The GCs were cultured in 24-well plates and transfected with the successfully constructed vector for 48 hours, and finally the Dual-Glo Luciferase Assay Kit (Promega, USA) was used to test the relative luciferase activity.

### Western blot analysis

Total protein was isolated from the GC samples and the BCA Protein Assay Kit (Vigorous Bio-technology Beijing Co., Ltd., Beijing, China) was used to quantitate the amount of protein. Then, after denaturation by boiling with 5× protein sodium dodecyl sulfate-polyacrylamide gel electrophoresis (SDS-PAGE) loading buffer for 10 min, the protein samples were separated by SDS-PAGE and transferred onto nitrocellulose membrane. The membranes were incubated with anti-hamartin primary antibody (1:1,000; Biorbyt, San Francisco, CA, USA). Following incubation with the secondary antibody for 1 hour at room temperature, the ECL-PLUS kit (Amersham Biosciences, Piscataway, NJ, USA) was used to visualize antibody-bound protein bands. Among them, an anti-GAPDH antibody (1:3,000; Sigma, St. Louis, MO, USA) was used as an internal control. Finally, ImageJ software was used to calculate the gray value of the band and the relative protein expression level of *PIK3R2* was normalized by GAPDH value.

### Data analysis

All statistical analyses were performed with R software and data were presented as means±standard deviation from at least three independent experiments. The significance of differences in means between two groups was analyzed by using Student’s t-test (two-tailed). * indicates p<0.05; ** indicates p<0.01.

## RESULTS

### miR-126-3p targets at *PIK3R2* and inhibits its mRNA and protein expression

We found *PIK3R2* was a target gene of miR-126-3p by using three bioinformatics algorithms, TargetScan, miRanda, and RNAhybrid (Figure 1B). To further confirm whether miR-126-3p was targeting *PIK3R2*, we co-transfected the constructed recombinant vectors PIK3R2-WT, PIK3R2-mutant (MUT) and PIK3R2-Del (Figure 1C) into GCs (Figure 1A) with miR-126-3p mimic or mimic NC, respectively. As shown in Figure 1D, we found that the luciferase activity of the miR-126-3p mimics in *PIK3R2*-WT was significantly lower than mimic NC (p<0.05), but the luciferase activity of the miR-126-3p mimics in *PIK3R2*-MUT and *PIK3R2*-Del showed no significant difference with mimic NC in GCs (Figure 1D). These results indicated that miR-126-3p inhibited the luciferase activity of the reporter gene by binding to the 3′UTR of *PIK3R2*.

To further explore the effects of miR-126-3p on the expression of *PIK3R2*, miR-126-3p mimic or miR-126-3p inhibitor was transfected into porcine GCs. Compared with mimic NC, miR-126-3p mimic significantly down-regulated the mRNA (Figure 1E, p<0.05) and protein (Figure 1G) level of *PIK3R2*. Compared with inhibitor NC, miR-126-3p inhibitor significantly up-regulated the mRNA (Figure 1F, p<0.05) and protein (Figure 1H) levels of *PIK3R2*. These observations indicated that miR-126-3p may target *PIK3R2* 3′UTR and repress its mRNA and protein expression level in porcine GCs.

### Knockdown *PIK3R2* inhibits GCs apoptosis and promotes GCs proliferation

**Figure 2:**
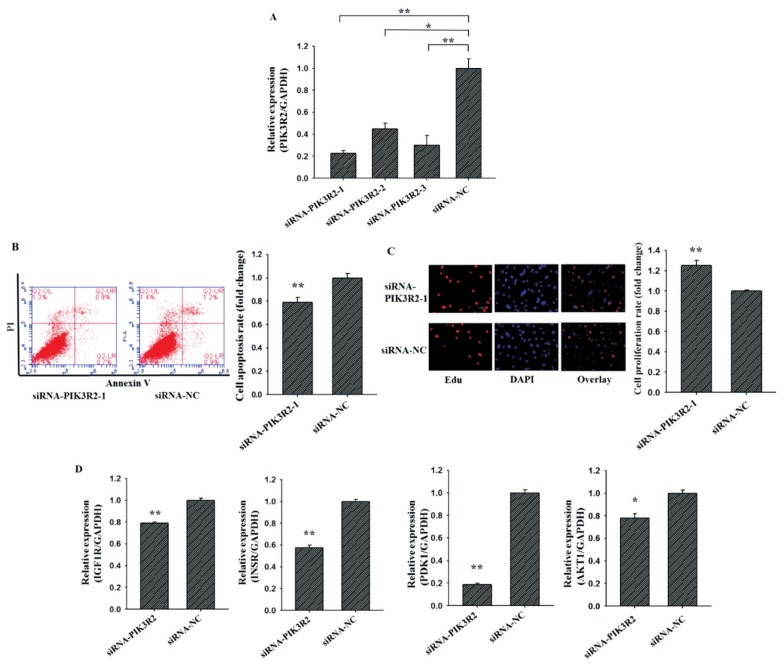
Knockdown *PIK3R2* might inhibit GCs apoptosis and promote GCs proliferation by disturbing PI3K pathway. SiRNA-*PIK3R2*-1 exhibited the best inhibition efficiency of *PIK3R2* by using quantitative RT-PCR (A). Knockdown *PIK3R2* could inhibit GCs apoptosis by using Annexin-V/PI staining and flow cytometry analysis (B). Knockdown *PIK3R2* could promote GCs proliferation by using EdU assays (C). Relative mRNA expression of several key genes of PI3K pathway, *IGF1R*, *INSR*, *PDK1*, and *AKT1*, after transfection with the *PIK3R2* inhibitors (D). *PIK3R2*, phosphatidylinositol 3-kinase regulatory subunit 2; GCs, granulosa cells; RT-PCR, real-time polymerase chain reaction; *IGF1R*, insulin-like growth factor 1 receptor; *INSR*, insulin receptor; *PDK1*, pyruvate dehydrogenase kinase 1; *AKT1*, serine/threonine kinase 1. * indicates p<0.05 and ** indicates p<0.01. Mimic NC is the negative control of miR-126-3p mimic and inhibitor NC is the negative control of miR-126-3p inhibitor. Compared with control, the fold change of GCs proliferation or apoptosis rate is presented in Figure 2B and 2C.

To investigate the cellular function of *PIK3R2* on GCs apoptosis and proliferation, three *PIK3R2*-specific small interfering RNAs (siRNA) (siRNA-*PIK3R2-1*, siRNA-*PIK3R2*-2, siRNA-*PIK3R2*-3) and negative control (siRNA-NC) were transfected into GCs (Figure 2A). As shown in Figure 2A, siRNA-*PIK3R2*-1 exhibited the best inhibition efficiency, and thus siRNA-*PIK3R2*-1 was selected for knockdown *PIK3R2* in GCs. As shown in Figure 2, the GC apoptosis rate of the siRNA-PIK3R2 group was significantly lower than siRNA-NC group (Figure 2B, p<0.01), and the GC proliferation rate of the siRNA-*PIK3R2* group was significantly higher than siRNA-NC group (Figure 2C, p<0.01). Furthermore, several key genes of the PI3K pathway, insulin-like growth factor 1 receptor (*IGF1R*) [26], insulin receptor (*INSR*) [27], pyruvate dehydrogenase kinase 1 (*PDK1*) [28] and serine/threonine kinase 1 (*AKT1*) [29] were selected and detected to characterize biological functions of *PIK3R2*. We found that the mRNA expressions of *IGF1R* (Figure 2D, p<0.01), *INSR* (Figure 2D, p<0.01), *PDK1* (Figure 2D, p<0.01), and *AKT1* (Figure 2D, p<0.05) in siRNA-*PIK3R2* group were all significantly lower than that in siRNA-NC group. These observations demonstrated that knockdown *PIK3R2* may inhibit GC apoptosis and promote GC proliferation by disturbing the PI3K pathway.

### miR-126-3p regulates granulosa cells apoptosis and proliferation by targeting *PIK3R2*

**Figure 3:**
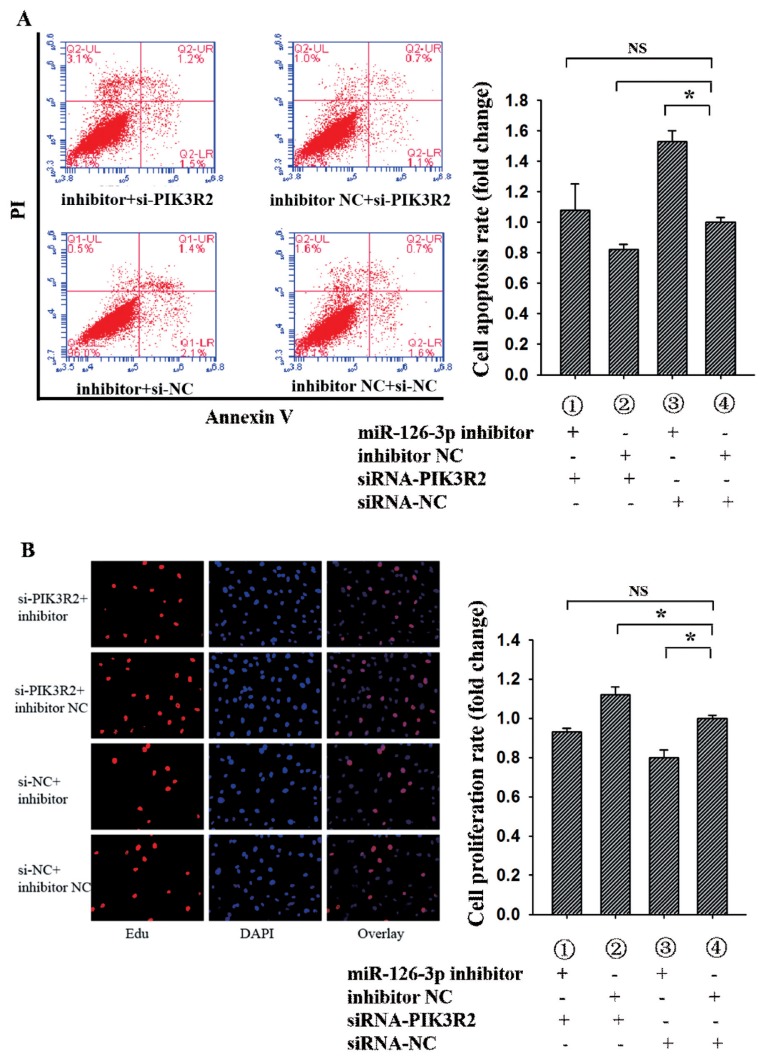
miR-126-3p inhibits GCs apoptosis and promotes GCs proliferation by targeting *PIK3R2*. Annexin-V/PI staining and flow cytometry analysis showed that miR-126-3p inhibited GCs apoptosis by targeting *PIK3R2* (A). EdU assays indicated that miR-126-3p promoted GCs proliferation by targeting *PIK3R2* (B). *PIK3R2*, phosphatidylinositol 3-kinase regulatory subunit 2; GCs, granulosa cells. * indicates p<0.05 and ** indicates p<0.01. Mimic NC is the negative control of miR-126-3p mimic and inhibitor NC is the negative control of miR-126-3p inhibitor. Compared with control, the fold change of GCs proliferation or apoptosis rate is presented in the Figure 3.

To further determine whether miR-126-3p inhibited GCs apoptosis and promoted GCs proliferation by targeting *PIK3R2*, miR-126-3p inhibitor, inhibitor NC, siRNA-*PIK3R2*, and siRNA-NC were co-transfected into porcine GCs. For the apoptosis rate of porcine GCs (Figure 3A), group 1 (miR-126-3p inhibitor + siRNA-*PIK3R2*) wasn’t notably different from group 4 (inhibitor NC + siRNA-NC) (p>0.05), group 2 (inhibitor NC + siRNA-*PIK3R2*) was significantly lower than group 4 (p<0.05), and group 3 (miR-126-3p inhibitor + siRNA-NC) was significantly higher than group 4 (p<0.05). These findings suggest that knockdown miR-126-3p could reverse the siRNA-*PIK3R2*-mediated inhibition of GC apoptosis, indicating that miR-126-3p may target *PIK3R2* to inhibit GCs apoptosis. For the proliferation rate of porcine GCs (Figure 3B), group 1 wasn’t notably different from group 4 (p>0.05), group 2 was significantly higher than group 4 (p<0.05), and group 3 was significantly lower than group 4 (p<0.05). These findings suggest that knockdown miR-126-3p could reverse the siRNA-*PIK3R2*-mediated promotion of GC proliferation, indicating that miR-126-3p may target at *PIK3R2* to promote GCs proliferation. The above results showed that miR-126-3p may inhibit GCs apoptosis and promote GCs proliferation by targeting *PIK3R2*.

## DISCUSSION

Previous evidence has indicated that miR-126-3p plays important roles in the process of folliculogenesis, oogenesis, and steroidogenesis in different species. In bovine corpus luteum, miR-126-3p is an important regulator of talin 2 and participates in luteal development during the estrous cycle [30]. In mouse, miR-126-3p can specifically inhibit progesterone receptor expression and β-casein secretion, then changes the viability of mammary epithelial cells and participates in mammary gland development [31]. In human primary ovarian GCs, miR-126-3p influences the mRNA level of apoptosis markers and suppresses progesterone, testosterone and estradiol secretion [32,33].

In our previous study, we have proved that miR-126-3p inhibits apoptosis and promotes proliferation of porcine ovarian GCs. In present study, the bioinformatics and luciferase activity assay showed that miR-126-3p was directly targeted *PIK3R2* 3′UTR. Moreover, miR-126-3p can negatively regulate the expression of *PIK3R2* at post-transcriptional and translational levels (Figure 1). siRNA-mediated *PIK3R2* knockdown could inhibit GC proliferation (Figure 3A) and promote GC apoptosis (Figure 3B), while miR-126-3p inhibitor abrogated these effects. These observations demonstrated that miR-126-3p could directly target *PIK3R2* and then inhibit apoptosis and promote proliferation of porcine ovarian GCs.

In this study, we found that siRNA-*PIK3R2* inhibited apoptosis (Figure 2B) and promoted proliferation (Figure 2C) of ovarian GCs. The result was in accordance with previous study where they found that knockdown *PIK3R2* could inhibit the apoptosis and promote the proliferation of rheumatoid arthritis synovial fibro-blasts cell. PI3K is a phosphatidylinositol-like compound which acts as the second messenger in the growth signaling pathway. Furthermore, studies have shown that PI3K signaling pathway may be involved in differentiation and proliferation of ovarian GCs, the selection and recruitment of luminal follicles, and the biological processes of mature follicle ovulation [34-36]. In this study, we also confirmed that interference with *PIK3R2* could significantly decrease expression levels of key genes on the PI3K signaling pathway, such as *IGF1R*, *INSR*, *PDK1*, and *AKT1*. Both *PDK1* and *AKT1* were positive regulators of primordial follicle activation, which stimulate the activation of the PI3K signaling pathway [37]. Studies have reported that when *PDK1* was knockout from oocytes of primordial follicles in mice, the majority of primordial follicles died directly from their dormant state around the onset of sexual maturity [38]. In *AKT1*-null mice, the number of growing antral follicles are reduced, while the number of degenerated oocytes are increased [39]. In *IGF1*-null mice, there are no mature large antral follicles produced and eventually resulting in infertile [40,41]. Compared with GCs of follicular cysts in cows, a recent study reported that *INSR* and PI3K were significantly higher expressed in GCs of control antral follicles. This indicated *INSR* and PI3K might involve in the regulation of steroidogenic enzymes expression [42].

**Figure 4:**
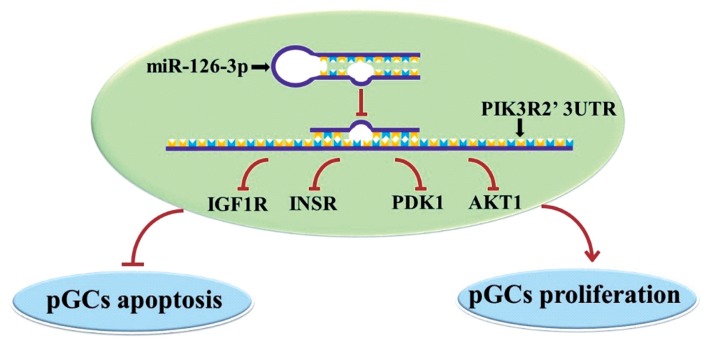
The potential regulatory modeling of miR126-3p and PIK3R2 in porcine GCs (pGCs). MiR-126-3p may target and inhibit the mRNA and protein expression of *PIK3R2*, thereby inhibiting several genes of PIK pathway, *IGF1R*, *INSR*, *PDK1*, and *AKT1*, which ultimately inhibit GCs apoptosis and promote GCs proliferation. *PIK3R2*, phosphatidylinositol 3-kinase regulatory subunit 2; GCs, granulosa cells; *IGF1R*, insulin-like growth factor 1 receptor; *INSR*, insulin receptor; *PDK1*, pyruvate dehydrogenase kinase 1; *AKT1*, serine/threonine kinase 1.

In conclusion, we found miR-126-3p could directly target at *PIK3R2* and inhibit apoptosis and promote proliferation of porcine ovarian GCs by down-regulating several key genes of the PI3K pathway, such as *IGF1R*, *INSR*, *PDK1*, and *AKT1* (Figure 4). This observation will help further understanding of the molecular mechanism of miR-126-3p function and its own expression regulation in porcine ovarian GCs.

## Figures and Tables

**Table 1:** Primers used in the present study

Name	Sequence	Product (bp)
qRT-PIK3R2-F	GACTGTGGGATTGAGACG	145
qRT-PIK3R2-R	ACCCGAGTAAGAATGTGC	
qRT-IGF1R-F	AAAGGAATGAAGTCTGGCTCC	209
qRT-IGF1R-R	TCAGCCTTGGAGATGAGCAG	
qRT-INSR-F	CACTGGCTATCGCATTGAGC	173
qRT-INSR-R	CCTGCCACATCAAGTGAACG	
qRT-PDK1-F	ACATGTACTCCACTGCACCC	91
qRT-PDK1-R	AGGCGTGATATGGGCAATCC	
qRT-AKT1-F	AGAAGCTCTTCGAGCTCATCCTCA	148
qRT-AKT1-R	TGCATGATCTCCTTGGCATCCTCA	
qRT-GADPH-F	GGACTCATGACCACGGTCCAT	220
qRT-GADPH-R	TCAGATCCACAACCGACACGT	
siRNA-PIK3R2-1	GGAGAAGUUACUUCAGGAA	
siRNA-PIK3R2-2	GGAACAACAAGCUGAUCAA	
siRNA-PIK3R2-3	GGUAUGUAGGCAAGAUCAA	
